# Biological control of *Erwinia mallotivora*, the causal agent of papaya dieback disease by indigenous seed-borne endophytic lactic acid bacteria consortium

**DOI:** 10.1371/journal.pone.0224431

**Published:** 2019-12-16

**Authors:** Mariam Dayana Mohd Taha, Mohammad Fahrulazri Mohd Jaini, Noor Baity Saidi, Raha Abdul Rahim, Umi Kalsom Md Shah, Amalia Mohd Hashim

**Affiliations:** 1 Department of Microbiology, Faculty of Biotechnology and Biomolecular Sciences, Universiti Putra Malaysia, Serdang, Selangor, Malaysia; 2 Department of Cell and Molecular Biology, Faculty of Biotechnology and Biomolecular Sciences, Universiti Putra Malaysia, Serdang, Selangor, Malaysia; 3 Department of Bioprocess Technology, Faculty of Biotechnology and Biomolecular Sciences, Universiti Putra Malaysia, Serdang, Selangor, Malaysia; Banaras Hindu University, INDIA

## Abstract

Dieback disease caused by *Erwinia mallotivora* is a major threat to papaya plantation in Malaysia. The current study was conducted to evaluate the potential of endophytic lactic acid bacteria (LAB) isolated from papaya seeds for disease suppression of papaya dieback. Two hundred and thirty isolates were screened against *E*. *mallotivora* BT-MARDI, and the inhibitory activity of the isolates against the pathogen was ranging from 11.7–23.7 mm inhibition zones. The synergistic experiments revealed that combination of *W*. *cibaria* PPKSD19 and *Lactococcus lactis* subsp. *lactis* PPSSD39 increased antibacterial activity against the pathogen. The antibacterial activity was partially due to the production of bacteriocin-like inhibitory substances (BLIS). The nursery experiment confirmed that the application of bacterial consortium *W*. *cibaria* PPKSD19 and *L*. *lactis* subsp. *lactis* PPSSD39 significantly reduced disease severity to 19% and increased biocontrol efficacy to 69% of infected papaya plants after 18 days of treatment. This study showed that *W*. *cibaria* PPKSD19 and *L*. *lactis* subsp. *lactis* PPSSD39 are potential candidate as biocontrol agents against papaya dieback disease.

## Introduction

Papaya (*Carica papaya* L.) is an economically significant tropical fruit grown in Malaysia. Papaya is widely cultivated because of their relevant economic and commercial impact [1] and has also been applied in traditional health applications [2]. The top papaya exporter in 2016 was Mexico at 169 kilotons (kt) or 47.3% of the global export, followed by Guatemala (13.8%), Brazil (10.6%) and Malaysia (6.9%). Papaya are exported to Europe, Hong Kong, Singapore and the Middle East [3,4]. The major limiting factor to papaya production in Malaysia is dieback disease caused by phytopathogenic bacteria, *Erwinia mallotivora*. The disease symptoms appeared as brown spots on the leaves, greasy spots and water-soaked lesions on the stem. Eventually severe infection causes the death of the papaya plant. The outbreak of the disease has caused enormous economic impact amounting to an estimated US$ 58 million due to a destruction of around 1 million trees and 200k metric tons of papayas [5,6]. Most papaya cultivars in Malaysia which include Eksotika, Sekaki and Setiawan are vulnerable to this pathogen [7].

The scarce availability of chemical treatments and absence of resistant papaya variety [7–9] have stimulated a growing interest in biological control agents (BCAs) for the management of papaya dieback disease. Biological control is a promising method to deliver lasting effect contributing to sustainable agriculture [10]. The application of endophytes as biocontrol agents has gained increasing attention as an alternative approach to control plant diseases. This is due to high similarity of ecological niche of endophytic bacteria to that of phytopathogen but does not induce any disease symptoms [11,12]. A small fraction of the endophytic microbiota of plants belongs to lactic acid bacteria (LAB) [13]. The LAB are known to have antagonistic abilities against pathogenic microbes and generally recognized as safe (GRAS) by the Food and Drug Administration (FDA, USA) [14,15], which make them ideal for applications in edible crop [16].

Previous works have proven that LAB strains from genus *Lactobacillus*, *Lactococcus* and *Weissella* can serve as biocontrol agents against bacterial and fungal phytopathogens [17–20]. LAB is considered as an excellent biocontrol agent for their ability to suppress pathogenic microorganisms through competition and antibiosis *via* the production of antimicrobial substances like bacteriocins and organic acids [21,22]. Application of a single inoculants might cause incoherent performance since a single biocontrol agent is not usually effective in whole agricultural ecosystems and all types of soil environment [23]. On the other hand, application of a combination of different bacteria might have greater effective control of papaya dieback disease than with single bacterial species inoculant.

Coinoculation is a way to combine different mechanism of different microbial species to increase plant performance and provide better biocontrol efficacy towards phytopathogens. The combination of multiple species in a consortium confers a more stable ecosystem as a result of increased beneficial interactions between several species as compared to single species [24]. Each individuals in the consortia communicates by exchanging signals or metabolites, subsequently work together to yield overall output [25]. This enables them to withstand a more complex or extreme environment, leading to better plant growth. Hence, the consortia world is seen as better option and has been growingly targeted for synthetic biology [25]. Better understanding in the microbe-microbe interactions will be useful for generating innovative ideas to manipulate them for human benefit, particularly as alternative to unsafe chemical pesticides to control plant diseases.

To the best of our knowledge, little is known about the activity of endophytic LABs in papaya plant and their use in papaya dieback disease control has not yet been reported. The aim of the current study was to find effective seed-borne endophytic LAB as biocontrol agents to suppress papaya dieback disease. The objectives of this study were to: (1) determine antagonistic and synergistic endophytic LAB isolated from papaya seed towards *E*. *mallotivora* BT-MARDI *in vitro*, (2) characterize the antibacterial substances produced by the selected endophytic LAB and (3) evaluate the control effects of single and combined endophytic LAB against papaya dieback disease under nursery condition.

## Materials and methods

### Culture and growth conditions

*Erwinia mallotivora* BT-MARDI strain was kindly provided by the Biotechnology Research Centre, Malaysian Agricultural Research and Development Institute (MARDI, Malaysia). The bacterial strain was cultivated on Luria Bertani (LB) agar, incubated at 28°C for 48 hr and stored at 4°C. For bacterial suspension, the bacterial pathogen was cultured in LB broth at 28°C for overnight with shaking (200 rpm)[6].

All lactic acid bacteria in the present study were grown at 30°C on MRS agar or MRS broth except when challenged with *E*. *mallotivora* BT-MARDI, which requires optimum growth temperature at 28°C.

### Fruits sampling and preparation of endophytic bacteria isolation

Papaya fruits were collected from 3 orchards located in Selangor and 1 in Perak, Malaysia and brought to the laboratory immediately for isolation of bacteria. Personal permission was granted from all independent orchard owners prior to sampling. The fruits were washed thoroughly with running water, surface sterilized with 70% (v/v) ethanol for 5 min, 2.5% (v/v) sodium hypochlorite solution (NaOCl) for 5 min and washed three times with sterile distilled water [26]. Under aseptic conditions, seeds and gelatinous sarcotesta were separated, crushed and macerated with 0.85% (w/v) sterilized saline solution (NaCl) using mortar and pestle to isolate seed-associated endophytic bacteria. The macerates were homogenized by vortexing at high speed for 60 seconds, aseptically tenfold diluted in sterile saline solution (0.85%, w/v) and cultured in de Man–Rogosa–Sharpe (MRS) broth (Oxoid, UK) at 30°C for overnight under aerobic condition without shaking to cultivate LAB strains. The enumerated endophytic bacteria were used further for preliminary screening.

### Isolation and *in vitro* screening of endophytic bacteria against *E*. *mallotivora* BT-MARDI

#### Preliminary screening

Antagonistic activity of endophytic bacteria strains was screened using a modified agar overlay method [27]. The bacterial suspensions were diluted to approximately 10^9^ CFU/mL. To perform agar overlay method, 100 μL of the diluted bacterial suspensions were spread on MRS agar (Oxoid, UK). *E*. *mallotivora* BT-MARDI served as the indicator strain was grown in LB broth at 28°C for overnight. The MRS agar was overlaid with 9 mL of LB soft agar (1% agar, w/v), seeded with 1 mL indicator bacterial suspension (approx. 10^9^ CFU/mL) and incubated under aerobic condition at 28°C for 48 h. Colonies with inhibition zone and distinct morphologies were randomly selected.

#### Secondary screening

**Fig 1:**
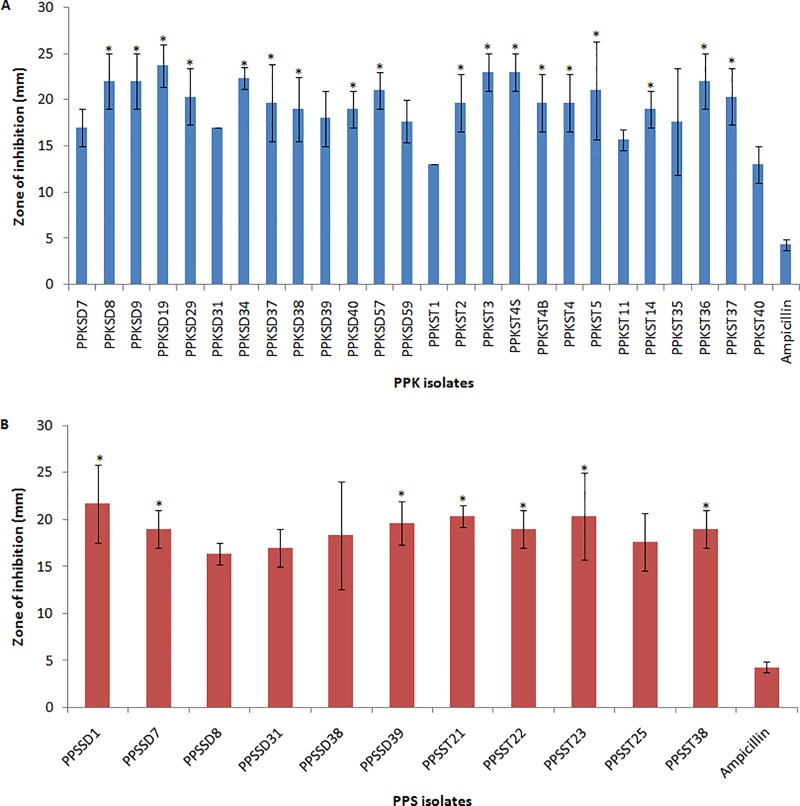
*In vitro* screening of endophytic bacteria for antagonistic activity using agar well diffusion method against *E*. *mallotivora* BT-MARDI, the causative agent of papaya dieback disease. (A) Inhibition zones formed by bacterial isolates isolated from papaya sample located in Perak (PPK); (B) Inhibition zones formed by bacterial isolates isolated from papaya sample located in Selangor (PPS). Values are means of three replications. Error bars show standard deviation. Bars marked with an asterisk indicate a significant antagonistic activity compared to positive control (Ampicillin) by Kruskal-Wallis test (N = 114, *p* < 0.05).

Previously selected bacterial colonies were assayed using the agar well diffusion method according to [28,29] with slight modifications. MRS agar was overlaid with LB soft agar containing indicator bacterial suspension in accordance with the preliminary screening. Wells with a diameter of 5 mm were cut per plate and the bottom of the wells was sealed with melted MRS medium. Antagonistic bacteria were cultured in MRS broth for overnight and 25 μL of the bacterial suspensions (approx. 10^9^ CFU/mL) was added into each well, with sterile MRS broth and ampicillin (128 mg/L) as negative and positive control, respectively. Three replicates per each isolate were tested. The plates were incubated at 28°C for overnight and zones of inhibition surrounding the well were measured. The potential isolates were sub-cultured on MRS agar to acquire pure cultures, and the antagonistic activity was reconfirmed using the same agar well diffusion method [28,29] (Fig 1). The purified strains were stored at -80°C in MRS broth supplemented with glycerol (20%, v/v) for further examination.

### Identification of antagonistic endophytic bacteria

#### Morphological and biochemical characterization

**Fig 2:**
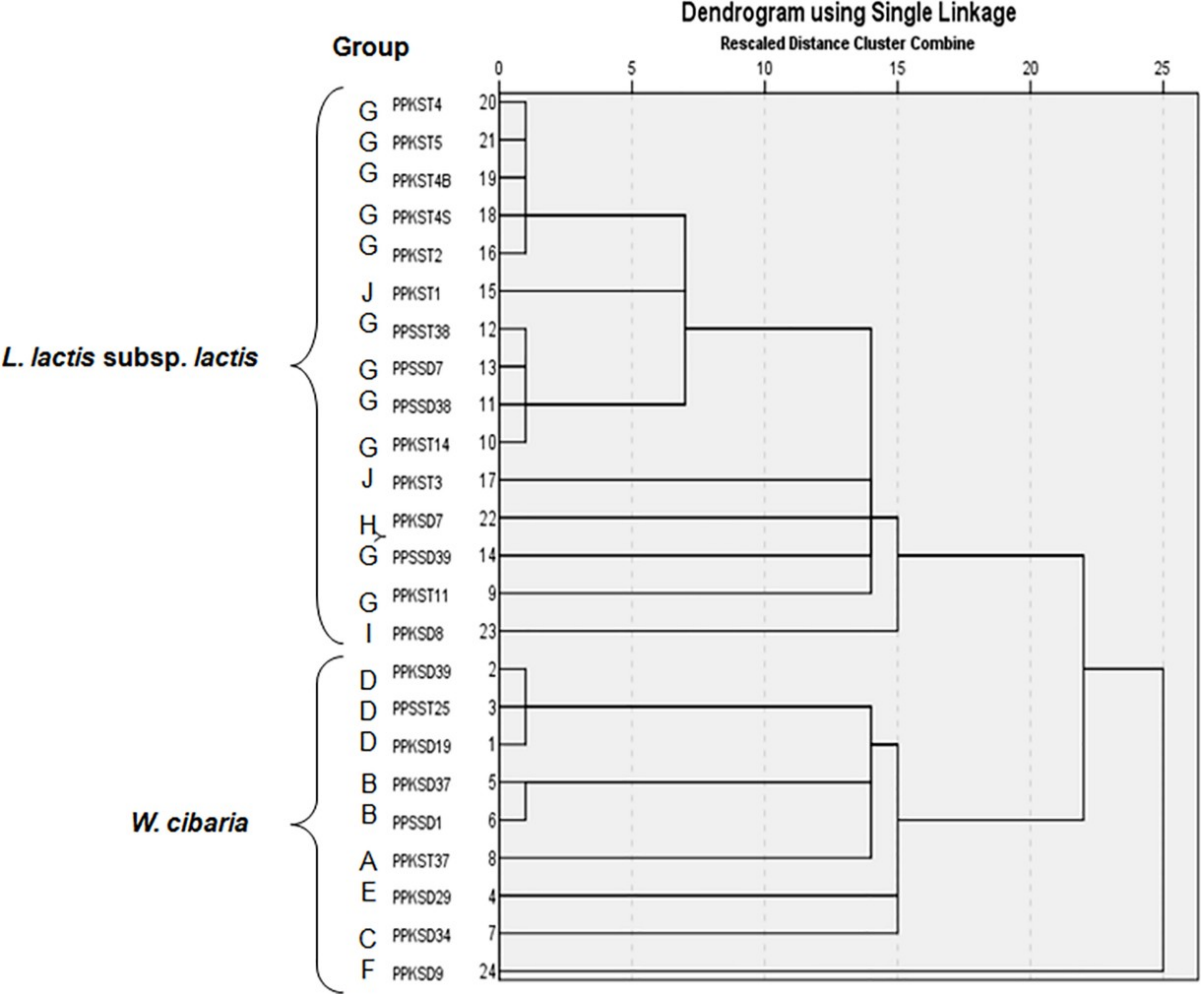
Dendrogram showing clustering and relationships of 24 potential isolates used in the study based on API 50 CH fermentation of 49 carbohydrates. The analysis was performed by calculating the Euclidean distance and the associations of isolates were constructed using the single linkage method (Nearest neighbour) in the IBM SPSS Statistic 22.0.

Bacterial colonies were subjected to morphological and biochemical tests which included Gram staining [30], catalase activity [31] and acidity test [32], and the results were summarized in S1 Table. Carbohydrate fermentation profiles were determined using API 50 CHL kit (Biomérieux, France) in accordance with the manufacturer’s instructions (S2 Table). A dendrogram was generated using Euclidean distance and single linkage method (Nearest neighbour) in the IBM SPSS Statistic 22.0 (Fig 2).

#### 16S rDNA gene sequencing analysis

**Fig 3:**
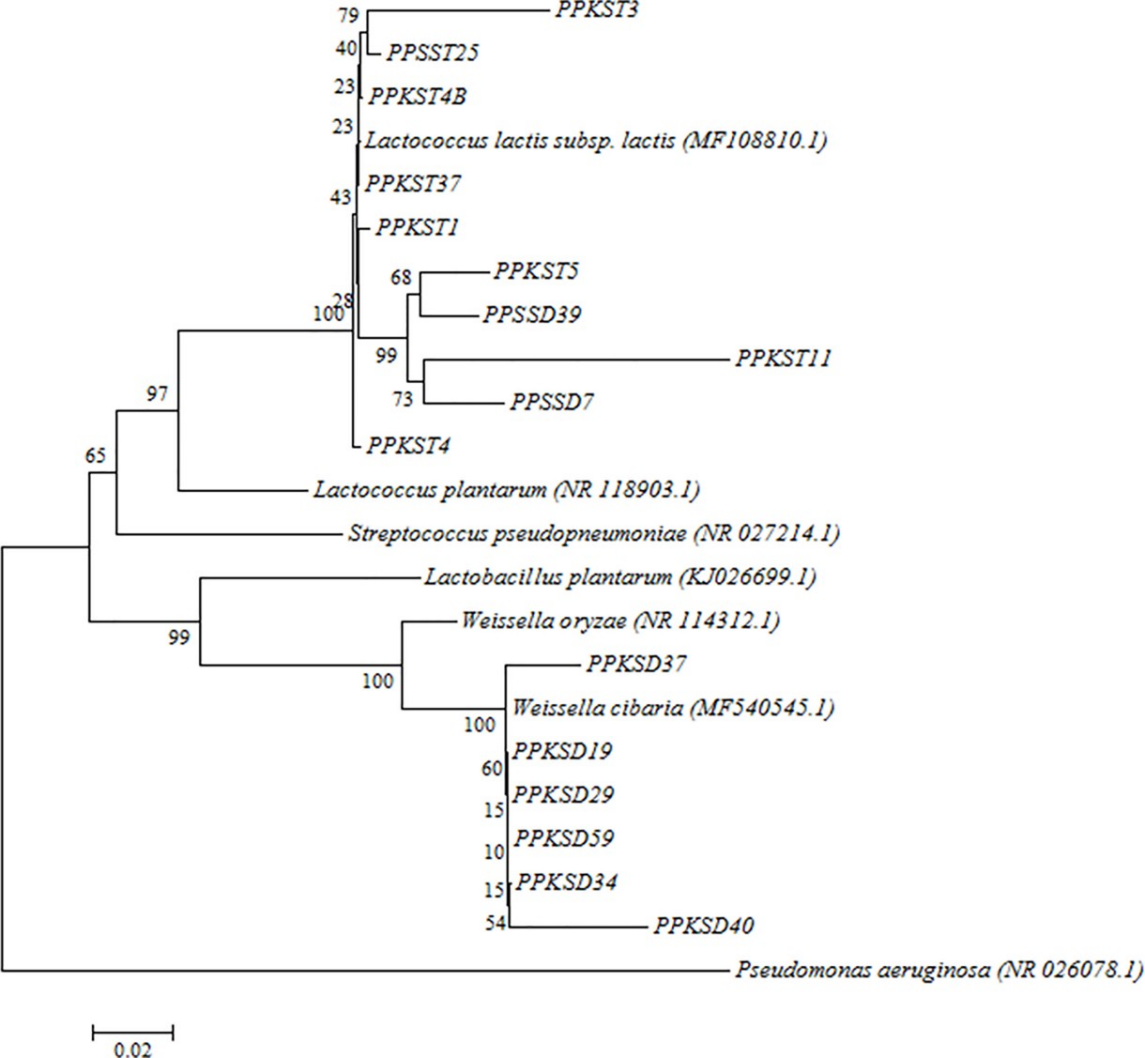
Phylogenetic tree showing the relative position of selected endophytic LAB isolates based on 16S rDNA partial sequences, using the Neighbor-Joining method. *Pseudomonas aeruginosa* was used as an outgroup. Bootstrap values of 1000 replications are displayed at the nodes of the tree, using MEGA 6. The scale bar corresponds to 0.02 units of the number of base substitutions per site. The GenBank accession numbers for nucleotide sequence data are shown in brackets.

The total genomic DNA of the antagonistic strains was extracted by Wizard genomic DNA purification kit (Promega, USA). The 16S rDNA genes of the isolates were amplified using 16S universal primers, 27F: (5'-AGAGTTTGATCCTGGCTCAG-3') and 1525R: (5'-AAGGAGGTGATCCAGCCGCA-3') [33]. The 1.5 kb amplified PCR products (S1 Fig) were purified using Wizard® SV Gel and PCR Clean-Up System Kit (Promega, USA) and sequenced by First BASE Laboratories Sdn. Bhd. (Selangor, Malaysia). Sequence data were edited and analyzed by FinchTV and basic local alignment search tool (BLAST) programs in the GenBank (NCBI). Phylogenetic tree was constructed by Neighbor-Joining method using ClustalW and MEGA 6 [34,35]. Grouping stability was calculated by 1000 bootstrap. The identity of the isolates was shown in Table 1 and the phylogenetic tree was depicted in Fig 3. The 16S rRNA sequence of an isolate, PPKSD19 was deposited in GenBank with accession number MN700179.

**Table 1:** Identification of endophytic LAB isolates isolated from papaya seeds using API 50 CH and 16S rDNA sequencing.

Tissue source	Bacteria	API identification	16S rDNA gene identification	Identities	GenBank Accession no.
Seed	PPKSD8	*L*. *lactis* subsp. *lactis* 1 (92.2%)	n.d	n.d	n.d
	PPKSD9	*W*. *confusa* (48.6%)	*W*. *cibaria* (96%)	413/428	MF540545.1
	PPKSD19	*W*. *confusa* (97.8%)	*W*. *cibaria* (100%)	1459/1459	MF540545.1
	PPKSD29	*W*. *confusa* (84.5%)	*W*. *cibaria* (100%)	1463/1463	MF540545.1
	PPKSD31	n.d	*W*. *cibaria* (94%)	412/438	MF540545.1
	PPKSD34	*W*. *confusa* (98.6%)	*W*. *cibaria* (99%)	1412/1414	MF540545.1
	PPKSD37	*W*. *confusa* (99.0%)	*W*. *cibaria* (100%)	1258/1258	MF540545.1
	PPKSD39	*W*. *confusa* (97.8%)	n.d	n.d	n.d
	PPKSD40	n.d	*W*. *cibaria* (97%)	1370/1413	MF540545.1
	PPKSD59	n.d	*W*. *cibaria* (100%)	1412/1412	MF540545.1
	PPSSD1	*W*. *confusa* (97.8%)	*W*. *cibaria* (99%)	364/365	MF540545.1
	PPKSD7	*L*. *lactis* subsp. *lactis* 1 (96.1%)	n.d	n.d	n.d
	PPSSD7	*L*. *lactis* subsp. *lactis* 1 (99.8%)	*L*. *lactis* subsp. *lactis* (99%)	1253/1258	MF108810.1
	PPSSD38	*L*. *lactis* subsp. *lactis* 1 (99.8%)	n.d	n.d	n.d
	PPSSD39	*L*. *lactis* subsp. *lactis* 1 (99.8%)	*L*. *lactis* subsp. *lactis* (99%)	1250/1253	MF108810.1
Sarcotesta	PPKST1	*L*. *lactis* subsp. *lactis* 1 (82.2%)	*L*. *lactis* subsp. *lactis* (99%)	1347/1348	MF108810.1
	PPKST2	*L*. *lactis* subsp. *lactis* 1 (99.8%)	n.d	n.d	n.d
	PPKST3	*L*. *lactis* subsp. *lactis* 1 (82.2%)	*L*. *lactis* subsp. *lactis* (96%)	1321/1383	MF108810.1
	PPKST4	*L*. *lactis* subsp. *lactis* 1 (99.8%)	*L*. *lactis* subsp. *lactis* (100%)	1372/1372	MF108810.1
	PPKST4S	*L*. *lactis* subsp. *lactis* 1 (99.8%)	*L*. *lactis* subsp. *lactis* (96%)	338/351	MF108810.1
	PPKST4B	*L*. *lactis* subsp. *lactis* 1 (99.8%)	*L*. *lactis* subsp. *lactis* (100%)	1417/1417	MF108810.1
	PPKST5	*L*. *lactis* subsp. *lactis* 1 (99.8%)	*L*. *lactis* subsp. *lactis* (100%)	1248/1248	MF108810.1
	PPKST11	*L*. *lactis* subsp. *lactis* 1 (99.8%)	*L*. *lactis* subsp. *lactis* (99%)	1115/1121	MF108810.1
	PPKST14	*L*. *lactis* subsp. *lactis* 1 (99.8%)	n.d	n.d	n.d
	PPKST37	*W*. *confusa* (99.6%)	*L*. *lactis* subsp. *lactis* (100%)	1418/1418	MF108810.1
	PPSST25	*W*. *confusa* (97.8%)	*L*. *lactis* subsp. *lactis* (99%)	1420/1430	MF108810.1
	PPSST38	*L*. *lactis* subsp. *lactis* 1 (99.8%)	n.d	n.d	n.d

n.d: Not determined

### Compatibility between antagonistic endophytic bacteria

**Fig 4:**
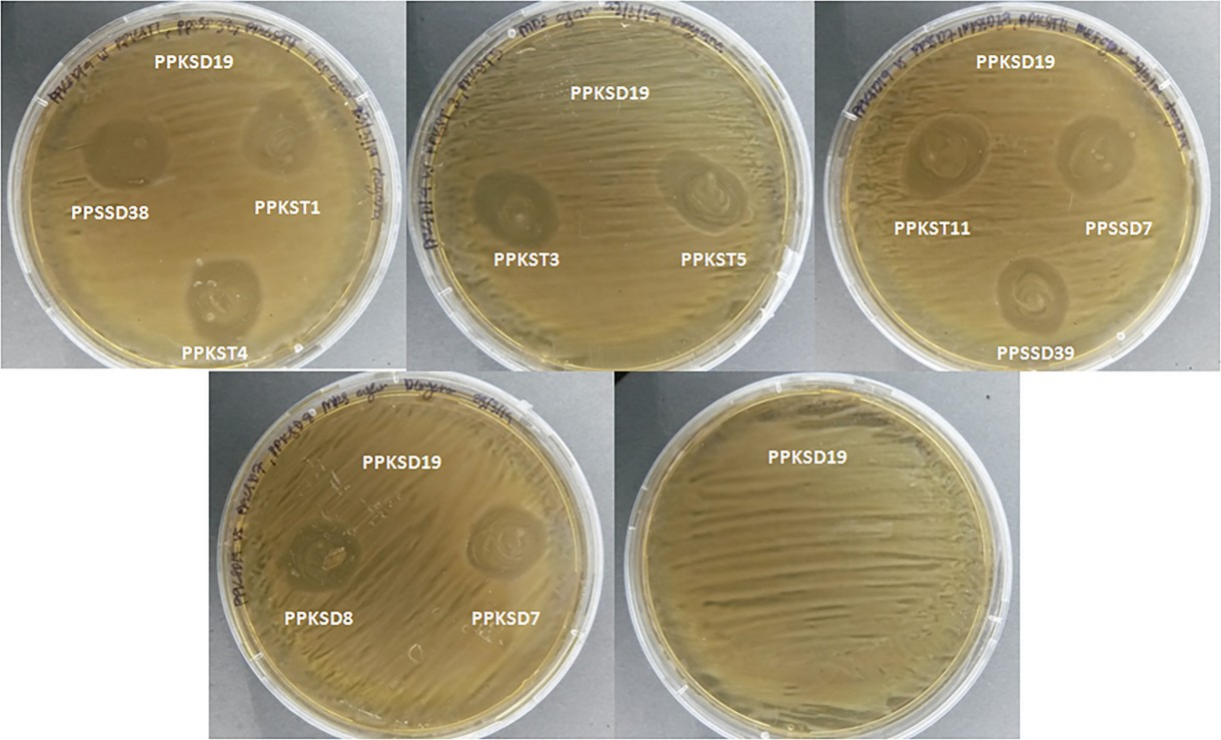
*In vitro* compatibility test between *W*. *cibaria* PPKSD19 and ten different isolates of *L*. *lactis* subsp. *lactis*. All *L*. *lactis* subsp. *lactis* isolates (PPSSD7, PPSSD38, PPSSD39, PPKSD7, PPKSD8, PPKST1, PPKST3, PPKST4, PKST5 and PPKST11) were streaked circularly while the *W*. *cibaria* PPKSD19 isolate was spread on the MRS agar plate.

Eleven bacterial antagonists PPKSD19, PPKSD7, PPKSD8, PPKST1, PPKST3, PPKST4, PPKST5, PPKST11, PPSSD7, PPSSD38 and PPSSD39 were tested for their compatibility among each other. The PPKSD19 isolate used as indicator was spread on MRS agar plates. Ten other tested strains were streaked circularly on the MRS agar plates. After incubation at 30°C for 48 h under aerobic conditions, the inhibition zones were observed. The presence of inhibition zones surrounding colonies indicates incompatibility of isolates. The compatibility tests were performed in triplicates (Fig 4).

### Synergistic effects of antagonistic endophytic bacteria against *E*. *mallotivora* BT-MARDI

The bacterial strains were tested individually and in combination for their *in vitro* synergistic interaction against indicator strain, *E*. *mallotivora* BT-MARDI according to [36] with slight modifications. By using agar-well diffusion assay, LB soft agar was inoculated with 10^9^ CFU/mL of indicator strain and subsequently was overlaid on MRS agar plates as described earlier. Wells of 5 mm diameter were filled with 25 μL of bacterial antagonist suspensions (approx. 10^9^ CFU/mL), individually. As for bacterial combination, a ratio of 1:1 (v/v) with 25 μL as the final volume was transferred into the wells. Three plates were prepared per treatment and incubated at 28°C for 48 h. After incubation, the inhibition zones due to individual and mutual effects of bacterial strains were measured (Table 2 and S2 Fig).

**Table 2:** Synergistic effect of *W*. *cibaria* and *L*. *lactis* subsp. *lactis* applied as single treatment or as consortia on inhibiting the growth of *E*. *mallotivora* BT-MARDI using agar well diffusion method.

Treatments^¥^	Inhibition zone (mm)^¶^	% increment over individual treatment^§^
PPKSD19	21.0±2.0^a^	n.a
PPKSD19 +PPSSD7	21.0±0.0^a^	n.c
PPKSD19 +PPSSD38	19.7±2.3^a^	- 6.2
PPKSD19 +PPSSD39	22.3±2.3^a^	6.2
PPKSD19 +PPKSD7	21.7±4.2^a^	3.3
PPKSD19 +PPKSD8	19.0±2.0^a^	- 9.5
PPKSD19 + PPKST1	15.7±3.1^b^	- 25.2
PPKSD19 + PPKST3	20.3±4.2^a^	- 3.3
PPKSD19 +PPKST4	16.3±3.1^b^	- 22.4
PPKSD19 + PPKST5	16.3±2.3^b^	- 22.4
PPKSD19 +PPKST11	19.7±1.2^a^	- 6.2
Ampicillin	3.7±0.6^c^	n.a

^¥^ Endophytic bacteria isolates applied were *W*. *cibaria* PPKSD19 and *L*. *lactis* subsp. *lactis* PPSSD7, PPSSD38, PPSSD39, PPKSD7, PPKSD8, PPKST1, PPKST3, PPKST4, PPKST5, PPKST11. Sterile MRS broth served as negative control and ampicillin as positive control.

^¶^ The diameter of the inhibition zone (mm) was calculated as radius from the outer edge of well multiplied by two (2r). Values are mean of three replications. Data are presented in the table as mean ± standard deviation.

In a column, means marked with different superscript letters indicate significant difference between treatments by Kruskal-Wallis test (N = 36, *p =* 0.026).

^§^ The increment percentage was calculated as (B-A)/Ax100, where A = inhibition diameter due to individual effect of *W*. *cibaria* PPKSD19, B = inhibition diameter due to a combined effect. Negative values indicate decreased inhibition effect.

n.a: Not applicable

n.c: No change in percentage compared to individual treatment.

### Verification of compatibility and synergistic activity of selected isolates

Compatibility between the selected isolates (PPKSD19 and PPSSD39) was reconfirmed using agar diffusion method as described by [37]. The isolates were cultured in MRS broth at 30°C overnight. The cell cultures were centrifuged at 6,000 rpm for 15 min. The cell pellets were adjusted to OD_600_:1.0 with fresh MRS broth. Wells of 10 mm were made on the centre of the MRS agar by using a sterile cork-borer. PPKSD19 was streaked by using sterile cotton bud onto the surface of MRS agar plate as indicator species. 100 μL of PPSSD39 culture was then pipetted into the MRS agar well. The test was performed vice versa by streaking PPSSD39 as indicator on MRS agar and challenged against PPKSD19 previously pipetted into the agar well. After 2 days of incubation at 30°C, the diameter of zone of inhibition was measured (S3 Fig).

Test for synergistic effect between PPKSD19 and PPSSD39 was demonstrated by agar well diffusion method on LB agar [37]. The method was slightly different than the method by [36] as described in the previous synergistic interaction test. Antagonists LAB were cultured in MRS broth whereas pathogenic *E*. *mallotivora* BT-MARDI as indicator species was cultured in LB broth and incubated at 28°C overnight. The overnight culture was centrifuged at 6,000 rpm for 15 min and the supernatant removed. The cell pellets were adjusted to OD_600_:1.0 with fresh MRS broth. Wells of 10 mm were made on the LB agar by using a sterile cork-borer. Then, *E*. *mallotivora* BT-MARDI was streaked by using sterile cotton bud onto the surface of LB agar plate. After a while, 100 μL of PPKSD19 alone, PPSSD39 alone, or the mixture of PPKSD19 and PPSSD39 (1:1 v/v) was pipetted into the LB agar well. After 2 days of incubation at 28°C, the zone of inhibition was measured (S3 Fig). Three replications were made for each treatment.

### Determination of antimicrobial substances

The production of antimicrobial substances was determined *in vitro* by critical dilution in an agar-well diffusion assay using Cell Free Supernatant (CFS) of bacterial strains PPKSD19 and PPSSD39 [38,39]. The bacterial strains were propagated in MRS broth for overnight at 30°C and the CFS were obtained by centrifugation (9500 x g, 10 min, 4°C). For the agar-well diffusion assay, MRS agar plates were overlaid with LB soft agar (1% agar, w/v) inoculated with 10^9^ CFU/mL of indicator strain, *E*. *mallotivora* BT-MARDI. Twenty five microlitres of CFS with initial acidic pH was adjusted to pH 6.5–7.0 (6N NaOH), two-fold diluted and added into the wells of 5 mm diameter made on the inoculated agar plates. After 48 h of incubation at 28°C, the diameter of inhibition zones was measured. The results were indicated as arbitrary units (AU/mL). One arbitrary unit (AU) was described as the reciprocal of the highest dilution that showed a clear zone of growth inhibition around the well [40]. Inhibitory activity due to hydrogen peroxide was determined by catalase (Sigma-Aldrich Corporation, USA) treatment of each pH-neutralized CFS for 1 h at 25°C at a final concentration of 1 mg/mL. The activity of antimicrobial substance was analyzed by treatment with proteinase K (Fisher Brand) at 37°C for 2 h with final concentration of 1 mg/mL. The thermal stability of the antimicrobial substances was tested by boiling the treated CFS at 100°C for 20 min. The results were indicated in Table 3. The residual antimicrobial activity of the CFS was measured as follows:

$$AU/mL=\frac{1000}{V}\;D$$

where *D* is the dilution factor and *V* is the volume of CFS.

**Table 3:** The effect of antibacterial substance produced by two LAB isolates with protease and heat treatment on inhibitory activity against *E*. *mallotivora* BT-MARDI.

Isolate	BLIS activity (AU/mL)^¥^	Inhibition zone values (mm)^Ω^					
		Control^¶^	pH-neutralized^§^	pH-neutralized, catalase-treated^‡^	Enzyme treatment		Heat treatment 100°C
					Control^†^	Proteinase K	
*W*. *cibaria* PPKSD19	40	11.8 ± 2.3^a^	11.2 ± 2.1^a^	6.2 ± 3.5^a^	+	-	6.2 ± 3.5^a^
	20	11.5 ± 2.6^a^	6.8 ± 4.0^ab^	2.7 ± 2.4^b^	+	-	2.7 ± 2.4^b^
	10	10.8 ± 5.0^a^	8.8 ± 1.2^a^	1.5 ± 2.6^b^	+	-	1.5 ± 2.6^b^
*L*. *lactis* subsp. *lactis* PPSSD39	40	6.8 ± 4.0^a^	6.2 ± 0.6^a^	3.2 ± 0.6^a^	+	-	3.2 ± 0.6^a^
	20	6.8 ± 0.6^a^	7.5 ±3.0^a^	2.7 ± 2.8^a^	+	-	2.7 ± 2.8^a^
	10	5.5 ± 2.0^a^	4.8 ± 1.2^a^	2.0 ± 1.8^a^	+	-	2.0 ± 1.8^a^

^¥^ BLIS activity was calculated as follows: AU/mL = (1000/*V*)*D, where *D* is the dilution factor and *V* is the volume of CFS.

^¶^ Cell free supernatant (CFS) of LAB isolates without any treatment

^§^ CFS with pH neutralized to 6.5

^‡^ CFS with pH neutralized to 6.5 and catalase-treated

^†^ Control = pH neutralized, catalase-treated CFS without addition of proteinase K

^Ω^ Data are presented as mean ± standard deviation from three replicate experiments

In a row, means marked with different superscript letter indicate a significant inhibitory activity as determined by ANOVA (*p* < 0.05)

+ Inhibition zones present

- No inhibition zones present

### Biocontrol assays of the selected endophytic bacteria under nursery conditions

Three month-old papaya plants (cv. Sekaki) were planted in pots using non-sterilized soil under nursery conditions. The *E*. *mallotivora* BT-MARDI strain was inoculated at the stem of papaya plants using the method described by [7] with slight modifications. Stem of the plants were pricked with sterile needle to make wounds. Sterile piece of cottons, which previously wetted with 200 μL of the bacterial pathogen suspensions (1 x 10^9^ CFU/mL) were placed on top of the wounds. The efficacy of the selected bacterial consortium consisting of PPKSD19 and PPSSD39 to suppress papaya dieback disease was evaluated by applying the suspensions of single or a mixture of the two isolates (1:1, v/v) on the wounds that were made previously. After pathogen inoculation, 500 μL of the bacterial suspensions (1 x 10^9^ CFU/mL) were used to drench sterile pieces of cotton and placed on top of the wounded stems. The cottons were enfolded with parafilms to avoid dehydration of inocula. Control plants were treated similarly but with the same volume of sterile saline water or ampicillin (1 mg/mL) instead of the antagonists.

**Fig 5:**
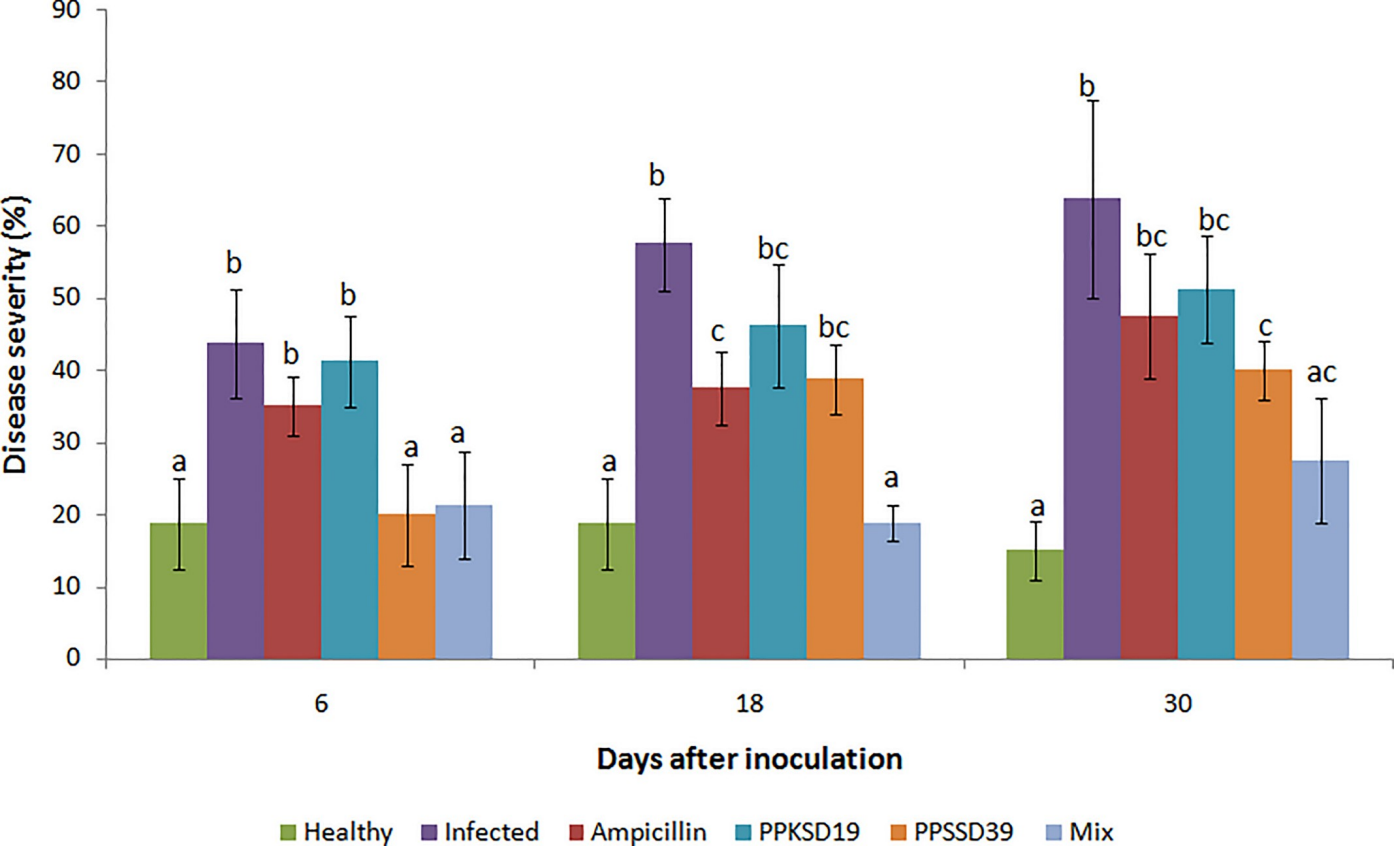
Disease severity of papaya dieback after treatment with single and mixture of *W*. *cibaria* PPKSD19 and *L*. *lactis* subsp. *lactis* PPSSD39. (A) 6^th^; (B) 18^th^; (C) 30^th^ day after inoculation with bacterial antagonists and *E*. *mallotivora* BT-MARDI. The data are the means of three replications per treatment with five plants per replication. Error bars show standard deviation of three replicates of each treatment. The means followed by different letters within a day of measurement indicate significant difference between treatments (ANOVA; *p* < 0.05).

**Fig 6:**
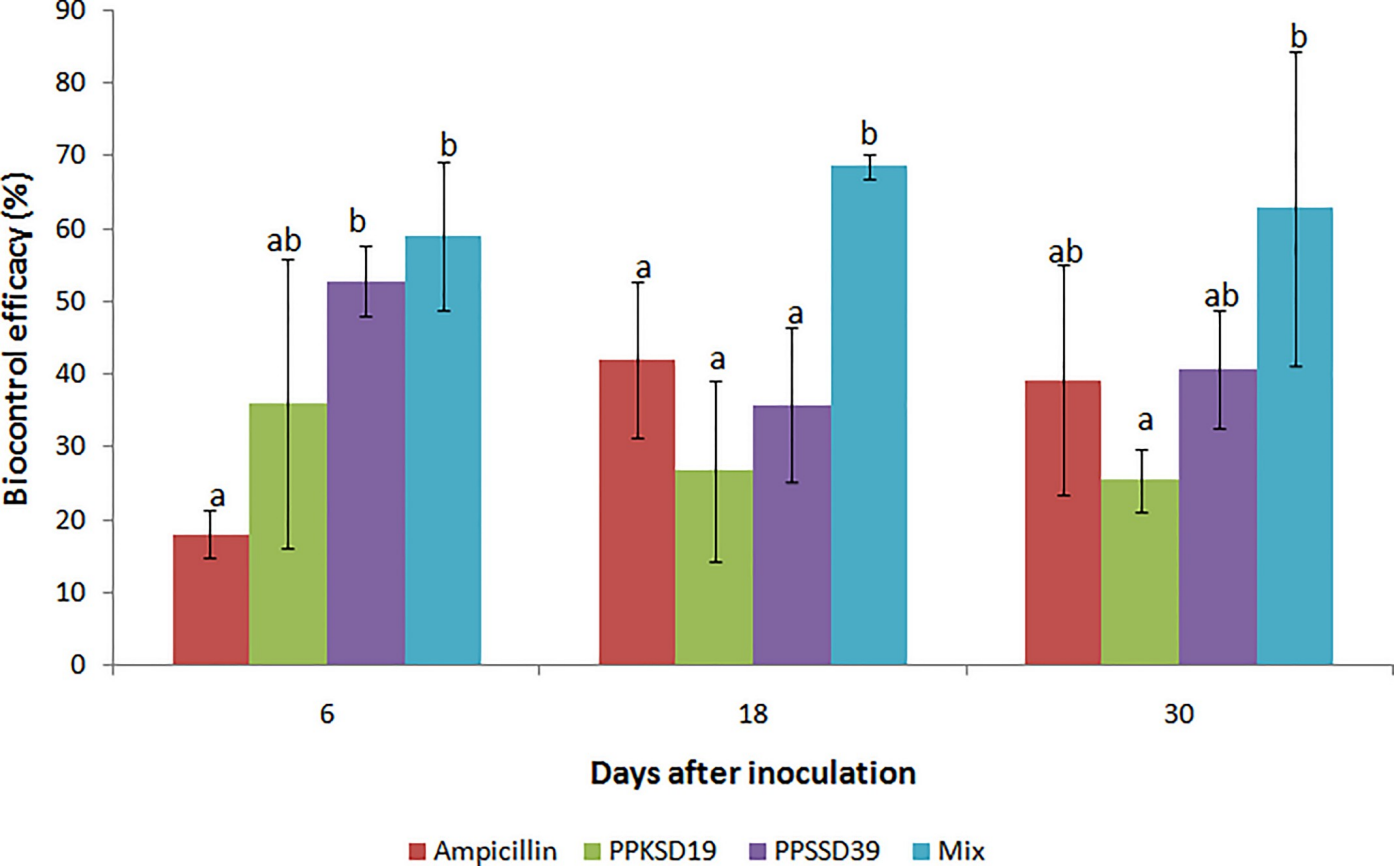
Biocontrol efficacy of papaya dieback after treatment with single and mixture of *W*. *cibaria* PPKSD19 and *L*. *lactis* subsp. *lactis* PPSSD39. (A) 6^th^; (B) 18^th^; (C) 30^th^ day after inoculation with bacterial antagonists and *E*. *mallotivora* BT-MARDI. The data are the means of three replications per treatment with five plants per replication. Error bars show standard deviation of three replicates of each treatment. The means followed by different letters within a day of measurement indicate significant difference between treatments (ANOVA; *p* < 0.05).

Six treatments were set in this study: (1) healthy control, (2) infected control, (3) ampicillin, (4) *W*. *cibaria* PPKSD19 treatment, (5) *L*. *lactis* subsp. *lactis* PPSSD39 treatment, (6) mixed treatment with *W*. *cibaria* PPKSD19 and *L*. *lactis* subsp. *lactis* PPSSD39 consortium. Un-inoculated plants without pathogen or antagonistic bacteria served as healthy control and plants inoculated with pathogen only served as infected control. The infected plants treated with bactericide ampicillin served as positive control. The experiment was carried out over 30-day period (from the day of pathogen inoculation) in a randomized complete block design (RCB) with six replicates of five plants each per treatment. The disease symptoms development was observed at six days intervals after inoculation based on the disease index (DI) scale as described by [41]: 0 = no wilt symptoms; 1 = < 25% of the leaves with wilt symptoms; 2 = 26–50% of the leaves with wilt symptoms; 3 = 51–75% of the leaves with wilt symptoms; 4 = > 76% of the leaves with wilt symptoms. Disease severity and biocontrol efficacy were determined (Figs 5 and 6 respectively) as follows:

$$Disease\;severity=\Sigma \;\frac{\left(Number\;of\;diseased\;plants\;in\;this\;index\times Disease\;index\right)}{\left(Total\;number\;of\;plants\;investigated\times The\;highest\;disease\;index\right)}\times 100$$

$$Biocontrol\;efficacy=\frac{(Disease\;severity\;of\;control\;plants–Disease\;severity\;of\;antagonist\;treated\;plants}{\left(Disease\;severity\;of\;control\right)}\times 100$$

### Statistical analysis

All univariate statistical analyses were conducted using SPSS v. 22.0 (IBM SPSS Inc., USA). Normality of the dataset was analyzed using the Shapiro-Wilk test. Nonparameter-wise means difference (*p* < 0.05) was analyzed by Kruskal-Wallis test, whereas One-way ANOVA using Tukey’s pairwise multiple comparison test (*p* < 0.05) was conducted for parametric dataset.

## Results

### Isolation and *in vitro* antagonistic activity of endophytic bacteria

In the preliminary screening, a total of 230 endophytic bacterial isolates were isolated from papaya seed samples of different sources (130 isolates from papaya samples collected in Selangor and 100 isolates from Perak, Malaysia). The colonies were selected based on distinct colony morphology and inhibitory effect against *E*. *mallotivora* BT-MARDI, which was evaluated using an *in vitro* agar overlay assay. Out of 230 bacteria, 37 endophytic bacterial isolates significantly inhibited the growth of *E*. *mallotivora* BT-MARDI (*p* < 0.05) compared to control in the second screening using an *in vitro* agar well diffusion method. The bacterial antagonists could inhibit the growth of the pathogen between 11.7 mm to 23.7 mm. Among these isolates, 14 isolates showed high antagonistic activity against *E*. *mallotivora* BT-MARDI with growth inhibition zone of >20 mm, and PPKSD19 displayed the greatest inhibition ability (23.7 mm) (Fig 1).

### Identification of antagonistic endophytic bacteria

The 37 isolates had large, grayish-white and small, creamy-white colonies, which were circular in shape on MRS agar plates (S1 Table). All potential isolates were Gram-positive short rods and cocci, catalase-negative and acid-forming bacteria. Based on these properties, the isolates were presumed as lactic acid bacteria. Twenty-four isolates were selected based on the strength of antagonistic activity and characterized by API 50 CH carbohydrate utilization pattern. All isolates (Group A to J) could ferment L-arabinose, D-xylose, D-glucose, D-fructose, D-mannose, n-acetyl-glucosamine, amygladin, arbutin, esculin ferric citrate, salicin, D-cellobiose, D-maltose, D-saccharose and gentiobiose, as carbon sources (S2 Table). Nine isolates (Group A to F) could not utilize 6 carbohydrates D-ribose, D-galactose, D-mannitol, D-lactose, D-trehalose, and amidon. The similarity of the carbohydrate utilization patterns was used to run a hierarchical cluster analysis (Fig 2). Cluster A, included 15 *Lactococcus lactis* subsp. *lactis* and cluster B composed of 9 *Weissella confusa* isolates.

The identification of isolates using the API 50 CHL showed a good agreement with 16S rDNA sequencing results, except for PPKSD9, PPKSD19, PPKSD29, PPKSD34, PPKSD37 and PPSSD1, which differed at the species level, and PPKST37 and PPSST2, which differed at the genus level (Table 1). The *Weissella* sp. mainly dominated the interior part of papaya seeds, whereas *Lactococcus* sp. was mainly detected in the sarcotesta. The 16S rDNA sequencing results identified PPKSD19, PPKSD29, PPKSD34, PPKSD37, PPKSD40 and PPKSD59 isolates as *W*. *cibaria* based on 97‒100% similarity to a GenBank entry with accession number MF540545.1. The isolates of PPKST1, PPKST3, PPKST4, PPKST4B, PPKST5, PPKST11, PPKST37, PPSSD7, PPSSD39 and PPSST25 that were identified as *L*. *lactis* subsp. *lactis* exhibited a similarity level of 96‒100% to a GenBank accession number MF108810.1, with 100% bootstrap support in the phylogenetic tree (Fig 3).

### Effect of mixtures of antagonistic endophytic bacteria on *E*. *mallotivora* BT-MARDI

The *W*. *cibaria* PPKSD19 and *L*. *lactis* subsp. *lactis* (PPSSD7, PPSSD38, PPSSD39, PPKSD7, PPKSD8, PPKST1, PPKST3, PPKST4, PPKST5 and PPKST11) were tested individually and in combination against *E*. *mallotivora* BT-MARDI for their *in vitro* synergistic interaction. All the treatments significantly inhibited the bacterial growth of the pathogen (*p* < 0.05) relative to control (Table 2 and S2 Fig). Combined application of PPKSD19 + PPSSD39 and PPKSD19 + PPKSD7 recorded a maximum inhibition zone of 22.3 mm and 21.7 mm with an increment of 6.2% and 3.3%, respectively. However, most of the consortia isolates showed a decrement in percentage (ranging from 3.3% to 25.2%) or no increment as compared to inhibitory activity of a single *W*. *cibaria* PPKSD19 strain.

### Compatibility among antagonistic bacteria and verification of synergistic activity of isolates

Ten different *L*. *lactis* subsp. *lactis* isolates PPSSD7, PPSSD38, PPSSD39, PPKSD7, PPKSD8, PPKST1, PPKST3, PPKST4, PPKST5 and PPKST11 were qualitatively observed for compatibility on MRS agar. All of the *L*. *lactis* subsp *lactis* isolates were inhibited by *W*. *cibaria* PPKSD19 indicating that the two genera were incompatible (Fig 4). Interestingly, they showed synergistic activity when tested against *E*. *mallotivora* despite their incompatibility (Table 2 and S2 Fig). Therefore, the synergistic activity of the incompatible PPKSD19 and PPSSD39 was further verified using a slightly different agar well diffusion method by [37] and the result was shown in S3 Fig. Similarly, the PPKSD39 and PPKSD19 inhibited each other in the absence of *E*. *mallotivora*. Intriguingly, despite incompatibility between both isolates, the PPKSD19-PPSSD39 consortium showed higher inhibition against the pathogen compared to the single culture treatment. Although there were slight variations in inhibition activity of individual isolates between results shown in Fig 1, Table 2 and S3 Fig, which might be due to different agar well diffusion method used or other experimental variations, the results consistently showed higher antagonistic activity of the mixed culture against *E*. *mallotivora* BT-MARDI as compared to the single inoculum treatment.

### Characterization of the antimicrobial substances

Due to the significant inhibition activity displayed by the combination of *W*. *cibaria* PPKSD19 and *L*. *lactis* subsp. *lactis* PPSSD39, the isolates were investigated for production of antimicrobial substances. The cell free supernatant (CFS) of the isolates could inhibit the growth of *E*. *mallotivora* BT-MARDI. The *W*. *cibaria* PPKSD19 exhibited a moderate but insignificant loss of activity (*p* > 0.05) when the CFS was neutralized at pH 6.5, demonstrating that the activity against the indicator strain was probably not related to the production of organic acids (Table 3).

A partial loss of activity by pH-neutralized CFS of *W*. *cibaria* PPKSD19 after catalase treatment indicated the hydrogen peroxide was partially responsible for the antimicrobial activity whereas the remaining activities may be due to other antimicrobial substances. However, the antimicrobial activity by *L*. *lactis* subsp. *lactis* PPSSD39 may neither be due to the production of hydrogen peroxide nor organic acid because the catalase-treated pH-neutralized CFS showed no significant reduction (*p* > 0.05). The antimicrobial activities of the pH-neutralized and catalase-treated CFS of both isolates were fully inactivated by proteinase K indicating the proteinaceous nature of the substance, which could be antimicrobial peptides. Since lactic acid bacteria are well-known to produce bacteriocins, both isolates are potentially secreting this substance.

The antimicrobial activity of the isolates was retained at 100°C for 20 min, suggesting that the proteinaceous compounds were heat-stable. Hence, it can be concluded that the antimicrobial activity of *W*. *cibaria* PPKSD19 on *E*. *mallotivora* BT-MARDI was mediated by synergistic action of hydrogen peroxide and bacteriocin-like inhibitory substances (BLIS), rather than organic acids. In contrast, the antimicrobial activity of *L*. *lactis* subsp. *lactis* PPSSD39 was likely due to the action of BLIS.

### Biocontrol effect of endophytic bacteria against papaya dieback disease *in planta*

The combination of *W*. *cibaria* PPKSD19 and *L*. *lactis* subsp. *lactis* PPSSD39 exhibited the highest antibacterial activity against *E*. *mallotivora* BT-MARDI. Hence, the efficacy of the single and consortia treatments of *W*. *cibaria* PPKSD19 and *L*. *lactis* subsp. *lactis* PPSSD39 in suppressing papaya dieback disease were evaluated by measuring disease severity of inoculated papaya plants (Fig 5). Plants inoculated with *E*. *mallotivora* BT-MARDI alone without bacterial antagonist exhibited 64% disease severity as observed for infected control plants at the 30^th^ day after inoculation. The biocontrol efficacy was high in the 6^th^ day of treatment by *L*. *lactis* subsp. *lactis* PPSSD39 at 53% but later reduced to 41% at the 30^th^ day of treatment (Fig 6). Meanwhile, treatment with *W*. *cibaria* PPKSD19 showed a relatively lower biocontrol efficacy than that of *L*. *lactis* subsp. *lactis* PPSSD39, at only 26% by day 30. Interestingly, disease severity was significantly reduced in plants treated with the bacterial mixture of *W*. *cibaria* PPKSD19 and *L*. *lactis* subsp. *lactis* PPSSD39. The treatment reduced disease severity by 19% and increased biocontrol efficacy by 69% after 18 days of pathogen challenge. This proved that disease suppression by the bacterial consortium *W*. *cibaria* PPKSD19 and *L*. *lactis* subsp. *lactis* PPSSD39 against papaya dieback disease was greater than the treatment with PPKSD19 or PPSSD39 alone.

## Discussion

In the present study, *W*. *cibaria* PPKSD19 and *L*. *lactis* subsp. *lactis* PPSSD39 were successfully isolated from the seed of papaya and effectively suppressed papaya dieback disease under nursery condition (Figs 5 and 6). LAB species are commonly used as bioprotective and biopreservatives agents of food due to their antagonistic features against foodborne pathogens [42,43], which make them ideal candidates for biocontrol agents against plant diseases. Nevertheless, reports on the use of LAB as biocontrol agents in plant protection are rather limited. To date, LAB are known to suppress few types of plant diseases such as bacterial soft rot [44,45], bacterial wilt [10], fire blight [43] and bean halo blight [46]. However, the potential of LAB in biological control of papaya dieback disease has not been reported.

The bacterial cultures on LAB-selective media showed that *W*. *cibaria* and *L*. *lactis* subsp. *lactis* were dominant in the papaya seeds. *W*. *cibaria* is commonly identified in many fermented foods, fruits and vegetables [47–49] while *L*. *lactis* subsp. *lactis* is present in dairy products and plant-based foods [50–53]. The competency of LAB to survive in the endosphere of a variety of plants suggests a profound plant–microbe interactions [54]. Seed-borne endophytes are of particular interest for their vertical transmission, ability to produce several antimicrobial compounds, enzymes, phytohormones and other secondary metabolites as well as ability to increase yield and biomass under abiotic and biotic stresses [55].

Previously, the antagonistic properties of *W*. *cibaria* from fresh fruits and vegetables was described against phytopathogenic bacteria, *Erwinia carotovora* and *Pseudomonas syringae* [20,56]. In the current study, the seed-borne *W*. *cibaria* PPKSD19 exhibited maximum *in vitro* antagonistic activity against *E*. *mallotivora* BT-MARDI in agar well diffusion assay with 23.7 mm inhibition diameter (Fig 1). The inhibitory effect on *E*. *mallotivora* BT-MARDI growth was likely due to synergistic interactions between *W*. *cibaria* PPKSD19 and *L*. *lactis* subsp. *lactis* PPSSD39 (Table 2, S2 Fig and S3 Fig). An important prerequisite for successful development of microbial consortia is the compatibility of the coinoculated microorganisms [57]. However, the results appear to contradict this notion. Both isolates were inhibiting each other, but became compatible when *E*. *mallotivora* BT-MARDI was present. This peculiar characteristic might be explained by microbe-microbe interactions inside the plant tissues that modulate the host reaction towards external stimuli. Microbes can communicate through secretion of signalling molecules or secondary metabolites that enable them to withstand an extreme or more complex environment [58]. We hypothesize that the pathogen might have relieved the antagonism or competition between *W*. *cibaria* PPKSD19 and *L*. *lactis* PPSSD39, either by degradation of the lethal compounds that both isolates may produced, or by exchanging secondary metabolites that assist the growth of both isolates [59], thus allowing them to co-exist and confer synergistic inhibitory activity against *E*. *mallotivora* BT-MARDI *in vitro* and *in planta*. Another plausible reason might be due different condition between *in vitro* and *in planta* experiments, as plants harbor more diverse microorganisms, hereby having more complex microbial interactions that probably better enhance or suppress the growth of certain interacting species. The mechanisms of synergistic activity of the duo in the presence of the pathogen remain to be investigated in future work, probably through metabolomic or proteomic studies. A few authors have proposed the combination of introduced biocontrol agents have to be compatible to improve disease control [60,61]. The enhancement of inhibitory activity of the antagonistic pairs may be due to the effective utilization of substrate which results in stimulation of the growth rate, production of nutrients by one bacterium that may be used by another and development of more balanced microbial community that may eliminate the pathogen [62]. In addition, the antimicrobial activities of LAB are not only due to the colonization and competition for nutrients and space, but also attributed to the production of diverse antimicrobial metabolites [44] as shown by the pair of antagonist in this study, *W*. *cibaria* PPKSD19 and *L*. *lactis* subsp. *lactis* PPSSD39.

The production of antimicrobial substances such as bacteriocins, organic acids, hydrogen peroxide, siderophores has been the primary mode of action for LAB in inhibiting the growth of pathogen, which can be exploited for biocontrol [10,63]. Based on the current findings, the potential antimicrobial substances produced by *W*. *cibaria* and *L*. *lactis* subsp. *lactis* that responsible for the inhibition of *E*. *mallotivora* BT-MARDI are most likely to be hydrogen peroxide and bacteriocin-like inhibitory substances (BLIS). Generally, antimicrobial compounds are believed to act synergistically to inhibit the growth of bacterial pathogen and eventually lead to cell death [64]. From the *in vitro* test, hydrogen peroxide was detected from *W*. *cibaria* PPKSD19 and partially effective against *E*. *mallotivora* BT-MARDI (Table 3). Similar observation was reported by Trias [20] where hydrogen peroxide produced by strains *W*. *cibaria* BC48 and TM128 inhibited a phytopathogenic and spoilage bacteria, *E*. *carotovora*. The elimination of the entire antimicrobial activities after treatment with proteinase K demonstrated that antimicrobial compounds secreted by both isolates are proteinaceous in nature. Protease sensitivity is a key standard in the classification of antimicrobial metabolites, hence the compounds produced could be bacteriocins [65]. Similar to bacteriocins, nisin Z and weissellicin 110 produced by *L*. *lactis* subsp. *lactis* [66] and *W*. *cibaria* [67] were also reported to be sensitive to proteinase K. The BLIS produced by the isolates were heat-stable since their activities were retained even after boiling (100°C) for 20 min indicating either class I or II bacteriocins [68]. Srionnual [67] reported that the bacteriocin produced by *W*. *cibaria* 110 remained stable at 121°C for 15 min, similar to bacteriocin MK02R produced by *L*. *lactis* subsp. *lactis* MK02R [69].

Under the nursery conditions, *L*. *lactis* subsp. *lactis* PPSSD39 was effective against *E*. *mallotivora* BT-MARDI. Surprisingly, the effectiveness of *W*. *cibaria* PPKSD19 was reduced *in planta*, despite strong antagonism *in vitro*. This could be due to its poor endophytic competence. Antagonism expressed by a bacterium against a pathogen in culture media does not guarantee an efficient role in suppressing the pathogen in plant [70,71]. In addition, a single biocontrol agent is unlikely to be effective in all types of agricultural ecosystems [23]. This was observed in this study where the combination of *W*. *cibaria* PPKSD19 and *L*. *lactis* subsp. *lactis* PPSSD39 significantly reduced dieback disease and increased biocontrol efficacy over control treatment under nursery condition. The consortium is more likely to contain multiple antibacterial compounds as compared to single inoculants. The combined isolates may have interacted synergistically to reduce variability in the control efficacy [72], increase the spectrum of disease control [60] and provide more adaptability in mechanisms of action against pathogens [70]. In addition, they could exclude pathogenic species colonizing plant tissues exposed to infection [43,73] and induced plant immune response [10].

## Conclusions

In conclusion, *W*. *cibaria* PPKSD19 and *L*. *lactis* subsp. *lactis* PPSSD39 appears to be promising candidates for biocontrol agents of papaya dieback disease due to good antagonism against *E*. *mallotivora* BT-MARDI despite their individual incompatibility. The antibacterial activity was likely associated with the production hydrogen peroxide and bacteriocin-like inhibitory substances (BLIS). The bacterial consortium effectively reduced disease severity and increased biocontrol efficacy compared to single inoculum treatment. Further investigations have to be carried out to determine the predominant biocontrol mechanisms of *W*. *cibaria* PPKSD19 and *L*. *lactis* subsp. *lactis* PPSSD39 to suppress the disease as well as their interesting behavior in which two previous ‘political opponents’ (PPKSD19 and PPSSD39) get united to combat against the ‘invader’ (*E*. *mallotivora*) of their land (plant host). The mediator (probably small proteins or metabolites) of this interesting interaction remains to be discovered in future work.

## Supporting information

S1 Table Phenotypic characteristics of representative isolates isolated from papaya seeds. (DOCX)

S2 Table API 50 CH fermentation patterns of isolated endophytic LAB from papaya seeds. (DOCX)

S3 Table Data underlying figures and tables (A) Fig 1, (B) Fig 5, (C) Fig 6, (D) S3 Fig, (E) Table 2, (F) Table 3.
(XLSX)

S1 Fig 16S rDNA gene amplification of representative endophytic LAB isolates. Lane M, HyperLadder^™^ 1kb (Bioline, USA;; 200–10,037bp); (A) Lane 1 (PPKSD31), 2 (PPKSD34), 3 (PPKSD37), 4 (PPKSD40) and 5 (PPKSD59); (B) Lane 6 (PPSSD39), 7 (PPSST25), 8 (PPKSD19), 9 (PPKSD29), 10 (PPKST11) and 11 (PPKST37); (C) Lane 12 (PPSSD7), 13 (PPKST2), 14 (PPKST4B), 15 (PPKST4) and 16 (PPKSD8).
(TIF)

S2 Fig Antagonistic and synergistic activity of potential isolates against *E*. *mallotivora* BT-MARDI using agar well diffusion method. -ve: negative control (MRS broth).
(TIF)

S3 Fig Verification of compatibility between PPSSD39 and PPKSD19, and their synergism in inhibiting *E*. *mallotivora* BT-MARDI using agar well diffusion assay. (A) Inhibition zones formed between PPSSD39 and PPKSD19. PPSSD39 vs PPKSD19 (indicator species: PPSSD39) and PPKSD19 vs PPSSD39 (indicator species: PPKSD19) were shown at the left and right of the panel, respectively. Agar well diffusion photos corresponding to each treatment were shown at the top of each bar; (B) Inhibition zones formed when single culture PPSSD39 alone (control), PPKSD19 alone (control), or mixed culture of PPSSD39-PPKSD19 were tested against *E*. *mallotivora* BT-MARDI. Agar well diffusion photos corresponding to each treatment were shown at the top of each bar. Means marked with different letters indicate significant difference at *p* < 0.05 using Kruskal Wallis test. Error bars indicate standard deviation of four replicates of each treatment.
(TIFF)

S4 Fig The effects of single and combined biological control agents on papaya dieback under nursery conditions. Six treatments were set in this study: (1) Healthy control (only saline water); (2) Infected control (pathogen-inoculated); (3) Positive control (ampicillin); (4) PPKSD19 treatment (single strain *W*. *cibaria* PPKSD19); (5) PPSSD39 treatment (single strain *L*. *lactis* subsp. *lactis* PPSSD39); (6) Mix treatment (bacterial consortium *W*. *cibaria* PPKSD19 and *L*. *lactis* subsp. *lacti*s PPSSD39).
(TIF)
