# Supplementary material for: Biological control of Erwinia mallotivora, the causal agent of papaya dieback disease by indigenous seed-borne endophytic lactic acid bacteria consortium
Source: PLoS One. 2019 Dec 16;14(12):e0224431. doi: 10.1371/journal.pone.0224431 (PMC6913974; doi:10.1371/journal.pone.0224431)
Supplement: S1 Table — (DOCX) [file pone.0224431.s001.docx]

S1 Table. Phenotypic characteristics of representative isolates isolated from papaya seeds

| **Characteristic** | **Group A** | **Group B** | **Group C** | **Group D** | **Group E** | **Group F** | **Group G** | **Group H** | **Group I** | **Group J** |
| --- | --- | --- | --- | --- | --- | --- | --- | --- | --- | --- |
| No. of isolates | 1 | 2 | 1 | 3 | 1 | 1 | 11 | 1 | 1 | 2 |
| Morphology | Circular | Circular | Circular | Circular | Circular | Circular | Circular | Circular | Circular | Circular |
| Size | Big | Medium | Big | Medium | Big | Medium | Small | Small | Small | Small |
| Colour | Greyish white | Greyish white | Greyish white | Greyish white | Greyish white | Greyish white | Creamy white | Creamy white | Creamy white | Creamy white |
| Shape | Short rods | Short rods | Short rods | Short rods | Short rods | Short rods | Coccoid | Coccoid | Coccoid | Coccoid |
| Gram stain | + | + | + | + | + | + | + | + | + | + |
| Catalase | - | - | - | - | - | - | - | - | - | - |
| Acidity | + | + | + | + | + | + | + | + | + | + |

+, positive

-, negative
