# Supplementary material for: Biological control of Erwinia mallotivora, the causal agent of papaya dieback disease by indigenous seed-borne endophytic lactic acid bacteria consortium
Source: PLoS One. 2019 Dec 16;14(12):e0224431. doi: 10.1371/journal.pone.0224431 (PMC6913974; doi:10.1371/journal.pone.0224431)
Supplement: S2 Table — (DOCX) [file pone.0224431.s002.docx]

S2 Table. API 50 CH fermentation patterns of isolated endophytic LAB from papaya seeds

| **Test** | **Group A** | **Group B** | **Group C** | **Group D** | **Group E** | **Group F** | **Group G** | **Group H** | **Group I** | **Group J** |
| --- | --- | --- | --- | --- | --- | --- | --- | --- | --- | --- |
| Glycerol | - | - | - | - | - | - | - | - | - | - |
| Erythritol | - | - | - | - | - | - | - | - | - | - |
| D-Arabinose | - | - | - | - | - | - | - | - | - | - |
| L-Arabinose | + | + | + | + | + | W | + | + | + | + |
| D-Ribose | - | W | - | W | - | - | + | + | + | + |
| D-Xylose | + | + | W | + | + | W | + | + | + | + |
| L-Xylose | - | - | - | - | - | - | - | - | - | - |
| D-Adonitol | - | - | - | - | - | - | - | - | - | - |
| Methyl-βD-Xylopyranoside | - | - | - | - | - | - | - | - | - | - |
| D-Galactose | W | W | - | - | - | W | + | + | - | + |
| D-Glucose | + | + | + | + | + | + | + | + | + | + |
| D-Fructose | + | + | + | + | + | + | + | + | + | + |
| D-Mannose | + | + | + | + | + | + | + | + | + | + |
| L-Sorbose | - | - | - | - | - | - | - | - | - | - |
| L-Rhamnose | - | - | - | - | - | - | - | - | - | - |
| Dulcitol | - | - | - | - | - | - | - | - | - | - |
| Inositol | - | - | - | - | - | - | - | - | - | - |
| D-Mannitol | - | - | - | - | - | + | + | + | - | + |
| D-Sorbitol | - | - | - | - | - | - | - | - | - | - |
| Methyl-αD-Mannopyranoside | - | - | - | - | - | - | - | - | - | - |
| Methyl-αD-Glucopyranoside | - | - | - | - | - | - | - | - | - | - |
| N-AcetylGlucosamine | + | + | + | + | + | + | + | + | + | + |
| Amygdalin | + | + | + | + | + | + | + | + | + | + |
| Arbutin | + | + | + | + | + | + | + | + | + | + |
| Esculin ferric citrate | + | + | + | + | + | + | + | + | + | + |
| Salicin | + | + | + | + | + | + | + | + | + | + |
| D-Cellobiose | + | + | + | + | + | + | + | + | + | + |
| D-Maltose | + | + | + | + | + | + | + | + | + | + |
| D-Lactose | - | - | - | - | - | + | + | + | + | + |
| D-Melibiose | - | - | - | - | - | - | - | - | - | - |
| D-Saccharose | + | + | + | + | + | + | + | + | + | + |
| D-Trehalose | - | - | - | - | - | - | + | + | + | + |
| Inulin | - | - | - | - | - | - | - | - | - | - |
| D-Melezitose | - | - | - | - | - | - | - | - | - | - |
| D-Raffinose | - | - | - | - | - | - | - | - | - | - |
| Amidon | - | - | - | - | + | W | + | W | W | W |
| Glycogen | - | - | - | - | - | - | - | - | - | - |
| Xylitol | - | - | - | - | - | - | - | - | - | - |
| Gentiobiose | + | + | + | + | + | W | + | W | W | + |
| D-Turanose | - | - | - | - | - | - | - | - | - | - |
| D-Lyxose | - | - | - | - | - | - | - | - | - | - |
| D-Tagatose | - | - | - | - | - | - | - | - | - | - |
| D-Fucose | - | - | - | - | - | - | - | - | - | - |
| L-Fucose | - | - | - | - | - | - | - | - | - | - |
| D-Arabitol | - | - | - | - | - | - | - | - | - | - |
| L-Arabitol | - | - | - | - | - | - | - | - | - | - |
| Potassium Gluconate | + | + | + | + | + | W | W | - | W | W |
| Potassium 2-Ketoglucanate | - | - | - | - | - | - | - | - | - | - |
| Potassium 5-Ketoglucanate | - | - | - | - | - | - | - | - | - | - |

+, positive;

W, weakly positive;

-, negative
