# Supplementary figures and images for: Biological control of Erwinia mallotivora, the causal agent of papaya dieback disease by indigenous seed-borne endophytic lactic acid bacteria consortium
Source: PLoS One. 2019 Dec 16;14(12):e0224431. doi: 10.1371/journal.pone.0224431 (PMC6913974; doi:10.1371/journal.pone.0224431)

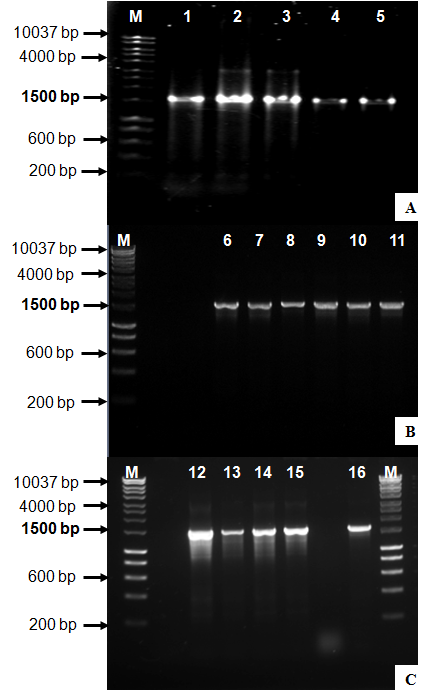

Supplement: S1 Fig — Lane M, HyperLadder™ 1kb (Bioline, USA;; 200–10,037bp); (A) Lane 1 (PPKSD31), 2 (PPKSD34), 3 (PPKSD37), 4 (PPKSD40) and 5 (PPKSD59); (B) Lane 6 (PPSSD39), 7 (PPSST25), 8 (PPKSD19), 9 (PPKSD29), 10 (PPKST11) and 11 (PPKST37); (C) Lane 12 (PPSSD7), 13 (PPKST2), 14 (PPKST4B), 15 (PPKST4) and 16 (PPKSD8). (TIF) [file pone.0224431.s004.tif]

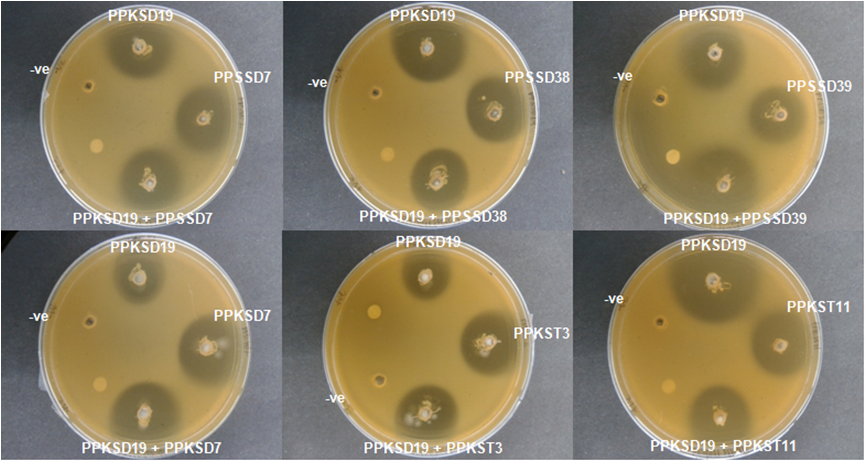

Supplement: S2 Fig — -ve: negative control (MRS broth). (TIF) [file pone.0224431.s005.tif]

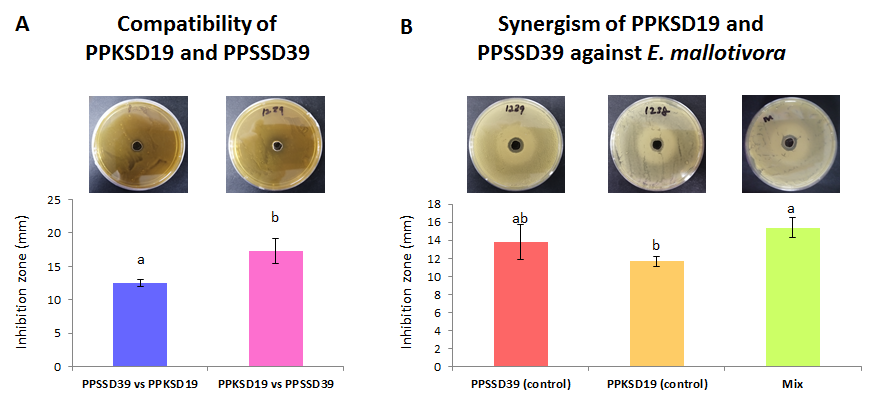

Supplement: S3 Fig — (A) Inhibition zones formed between PPSSD39 and PPKSD19. PPSSD39 vs PPKSD19 (indicator species: PPSSD39) and PPKSD19 vs PPSSD39 (indicator species: PPKSD19) were shown at the left and right of the panel, respectively. Agar well diffusion photos corresponding to each treatment were shown at the top of each bar; (B) Inhibition zones formed when single culture PPSSD39 alone (control), PPKSD19 alone (control), or mixed culture of PPSSD39-PPKSD19 were tested against E. mallotivora BT-MARDI. Agar well diffusion photos corresponding to each treatment were shown at the top of each bar. Means marked with different letters indicate significant difference at p < 0.05 using Kruskal Wallis test. Error bars indicate standard deviation of four replicates of each treatment. (TIFF) [file pone.0224431.s006.tiff]

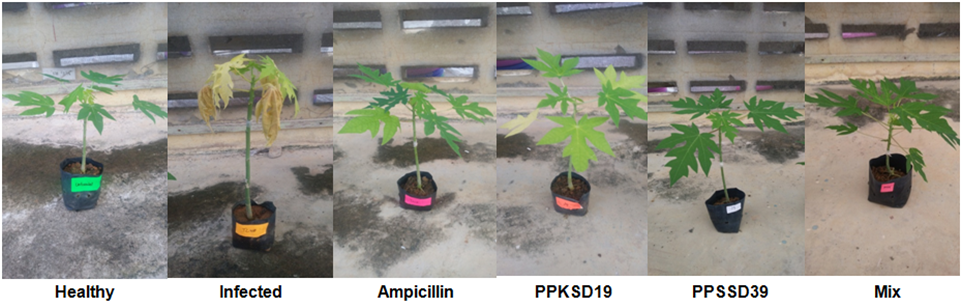

Supplement: S4 Fig — Six treatments were set in this study: (1) Healthy control (only saline water); (2) Infected control (pathogen-inoculated); (3) Positive control (ampicillin); (4) PPKSD19 treatment (single strain W. cibaria PPKSD19); (5) PPSSD39 treatment (single strain L. lactis subsp. lactis PPSSD39); (6) Mix treatment (bacterial consortium W. cibaria PPKSD19 and L. lactis subsp. lactis PPSSD39). (TIF) [file pone.0224431.s007.tif]
